# Supplementary material for: Over-Expression of Dehydroascorbate Reductase Improves Salt Tolerance, Environmental Adaptability and Productivity in Oryza sativa
Source: Antioxidants (Basel). 2022 May 28;11(6):1077. doi: 10.3390/antiox11061077 (PMC9220092; doi:10.3390/antiox11061077)
Supplement: Supplementary file 1 [file antioxidants-11-01077-s001.zip › antioxidants-1727472-supplementary.pdf]

# Supporting Information

## High Expression of Dehydroascorbate Reductase Improves Salt Tolerance, Environmental Adaptability, and Productivity in *Oryza sativa*

**Young-Saeng Kim [1,*], Seong-Im Park [2,†], Jin-Ju Kim [2], Sun-Young Shin [3], Sang-Soo Kwak [5], Choon-Hwan Lee [6], Hyang-Mi Park [7], Yul-Ho Kim [8], Il-Sup Kim [4,*] and Ho-Sung Yoon [2,3,4,*]**

[1] Research Institute of Ulleung-do & Dok-do, Kyungpook National University, Daegu 41566, Korea; kyslhh1228@hanmail.net
[2] School of Life Sciences, BK21 Plus KNU Creative BioResearch Group, Kyungpook National University, Daegu 41566, Korea; sheep91528@naver.com (S.-I.P.); deenamon@naver.com (J.-J.K.)
[3] Department of Biology, College of Natural Sciences, Kyungpook National University, Daegu 41566, Korea; snowmooa@hanmail.net
[4] Plant Systems Engineering Research Center, Korea Research Institute of Bioscience and Biotechnology, Daejeon 34141, Korea; sskwak@kribb.re.kr
[5] Department of Molecular Biology, Pusan National University, Busan 46241, Korea; chlee@pusan.ac.kr
[6] National Institute of Crop Science, Rural Development Administration, Jeonju 54875, Korea; parkhm2002@korea.kr
[7] Highland Agriculture Research Institute, National Institute of Crop Science, Rural Development Administration, Pyeongchang 25342, Korea; kimyuh77@korea.kr
[8] Advanced Bio-Resource Research Center, Kyungpook National University, Daegu 41566, Korea
* Correspondence: 92kis@hanmail.net (I.-S.K.); hsy@knu.ac.kr (H.-S.Y.)
† These authors contributed equally to this work.

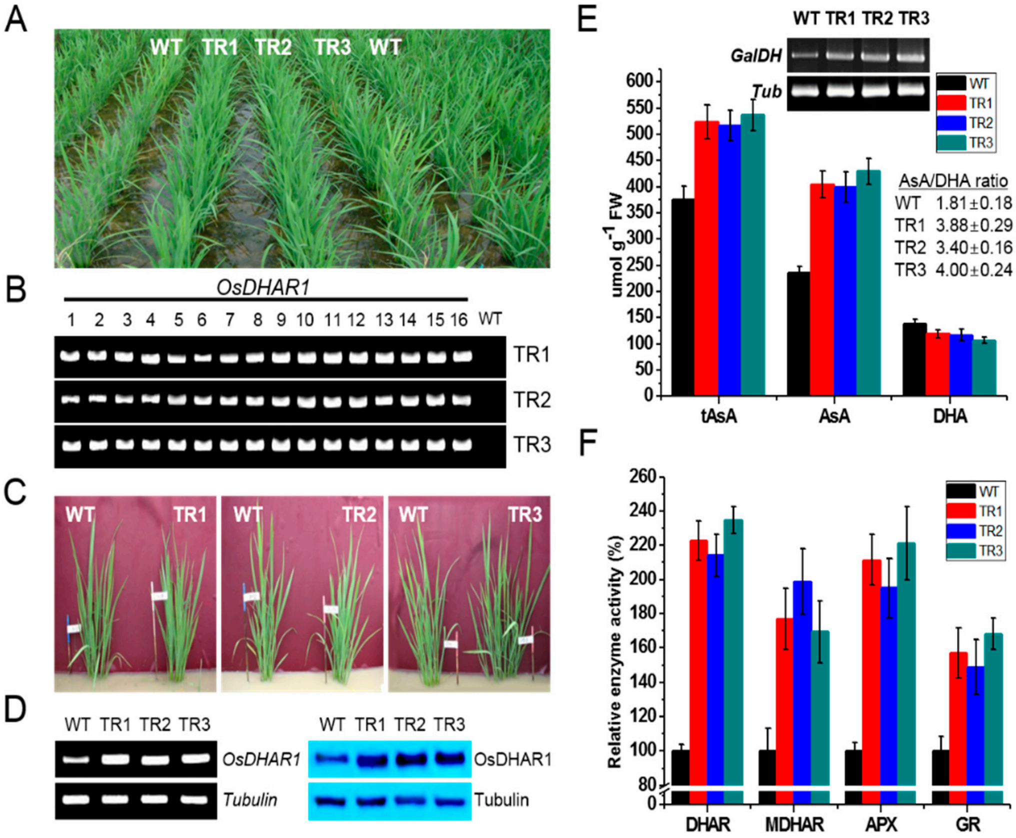

**Figure S1.** Environmental adaptability of *OsDHAR1*-expressing transgenic plants in a natural paddy field. (**a**) Phenotype analysis of TR and WT plants in the vegetative stage. (**b**) PCR confirmation of *OsDHAR1* expression in three separate TR plants (TR1-TR3). (**c**) Phenotype of TR and WT plants grown for 4 weeks in the paddy fields. (**d**) Confirmation of *OsDHAR1* expression using semi-quantitative RT-PCR and western blot analysis. Signal intensity in WT plants was due to endogenous *DHAR1* expression. *Tubulin* (*Tub*) and corresponding protein were used as loading controls. WT, wild-type rice; TR, transgenic rice. (**e**) Ascorbate pool and *GalDH* expression in TR and WT plants 4 weeks after transplantation. (**f**) Enzymatic activity of TR and WT plants grown for 4 weeks in the paddy field.

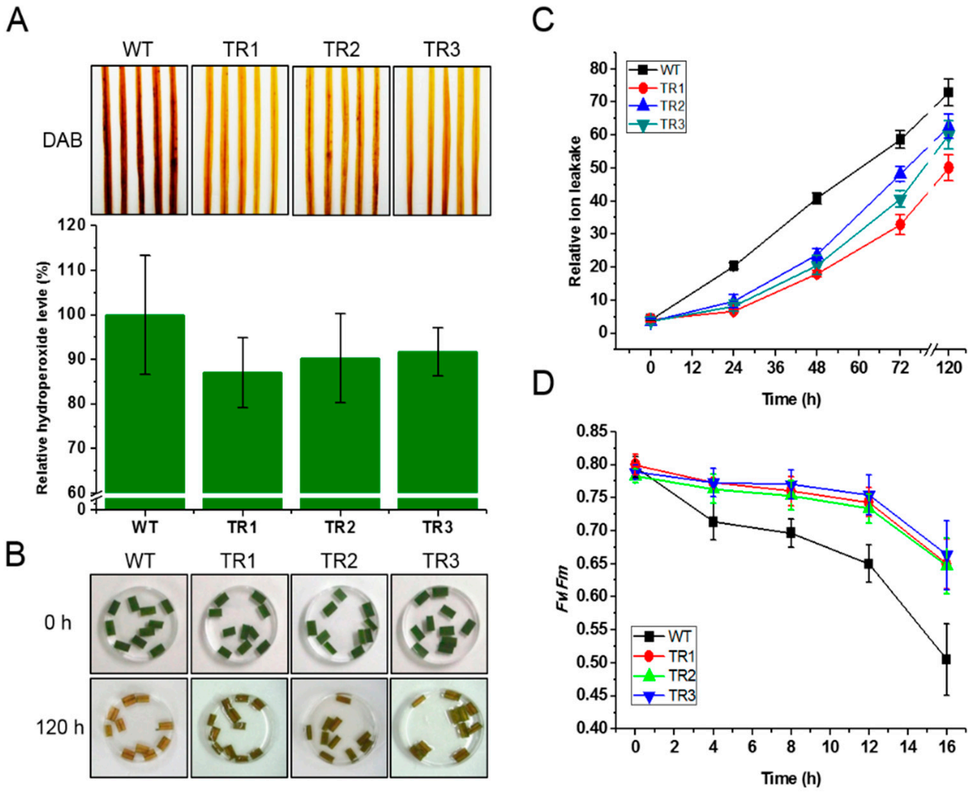


**Figure S2.** Redox state and photosynthetic capacity of *OsDHAR1*-expressing transgenic plants. (**a**) 3,3-Diaminobenzidine (DAB) staining phenotype and relative hydroperoxide level in leaves from TR and WT plants treated with 50 μM methyl viologen (MV) for 12 h. (**b**) Phenotype analysis of cells damaged in leaf discs of TR and WT plants exposed with 10 μM MV for 120 h. (**c**) Relative ion leakage in TR and WT plants treated with 10 μM MV from 0 h to 120 h. (**d**) Changes in chlorophyll fluorescence (*Fv*/*Fm*) in three independent TR and WT plants in response treatment with10 μM MV.

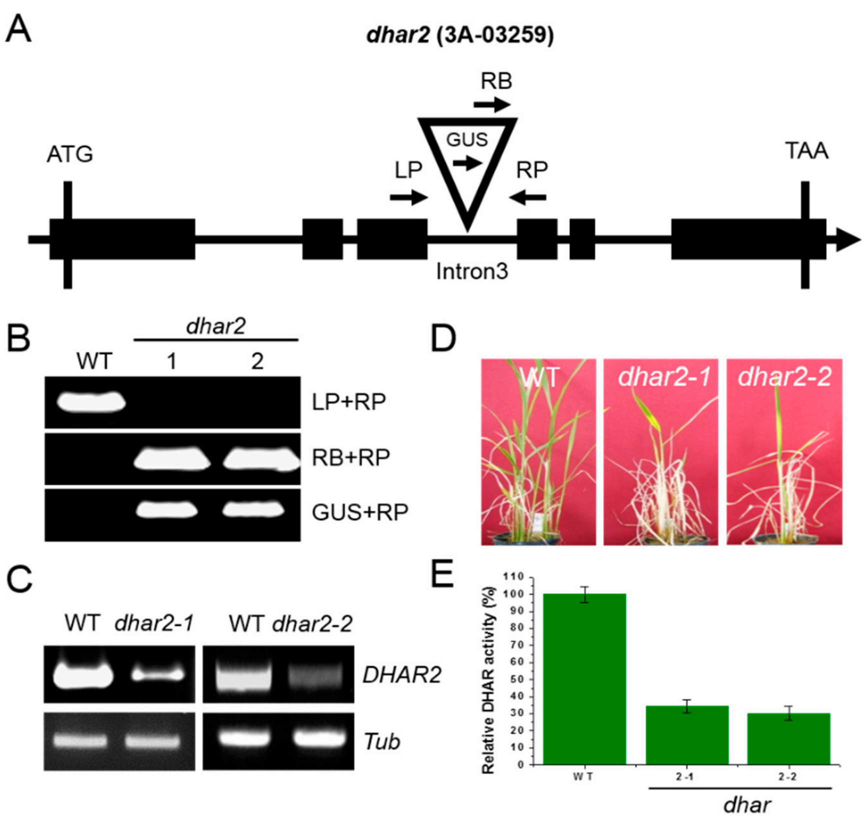


**Figure S3.** Characterization of *OsDHAR2* knockdown mutants. (**a**) Insertion positions of T-DNA spanning the mutation site in *dhar2* (3A-03259). (**b**) PCR-based confirmation of *dhar2* mutant lines. (**c**) Evaluation of *DHAR2* expression in *dhar2* plants using semi-quantitative RT-PCR. (**d**) Relative DHAR activity in *dhar2* and WT plants. (**e**) Phenotype of *dhar2* and WT plants treated with 100 mM NaCl for 25 days.

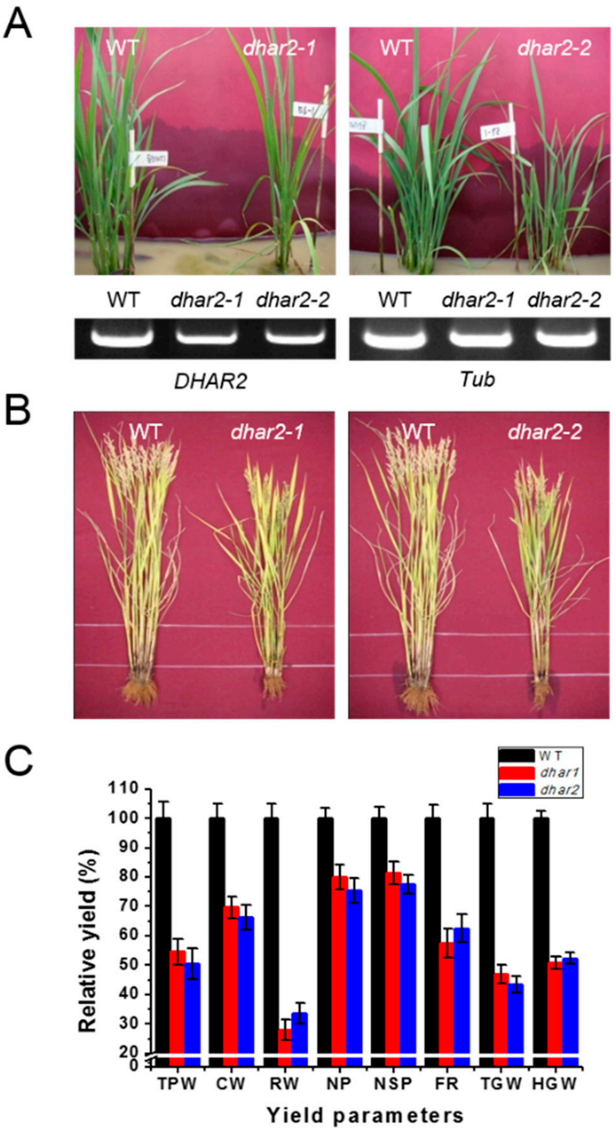


**Figure S4.** The poor the agronomic parameters of the *dhar2* plants grown in the paddy field. (**a**) Phenotype and *DHAR2* expression in *dhar2* and WT plants grown for 4 weeks in the paddy fields. (**b**) Phenotypes of *dhar2* and WT plants at the late reproductive stage. (**c**) Agronomic traits of *dhar2* and WT plants.

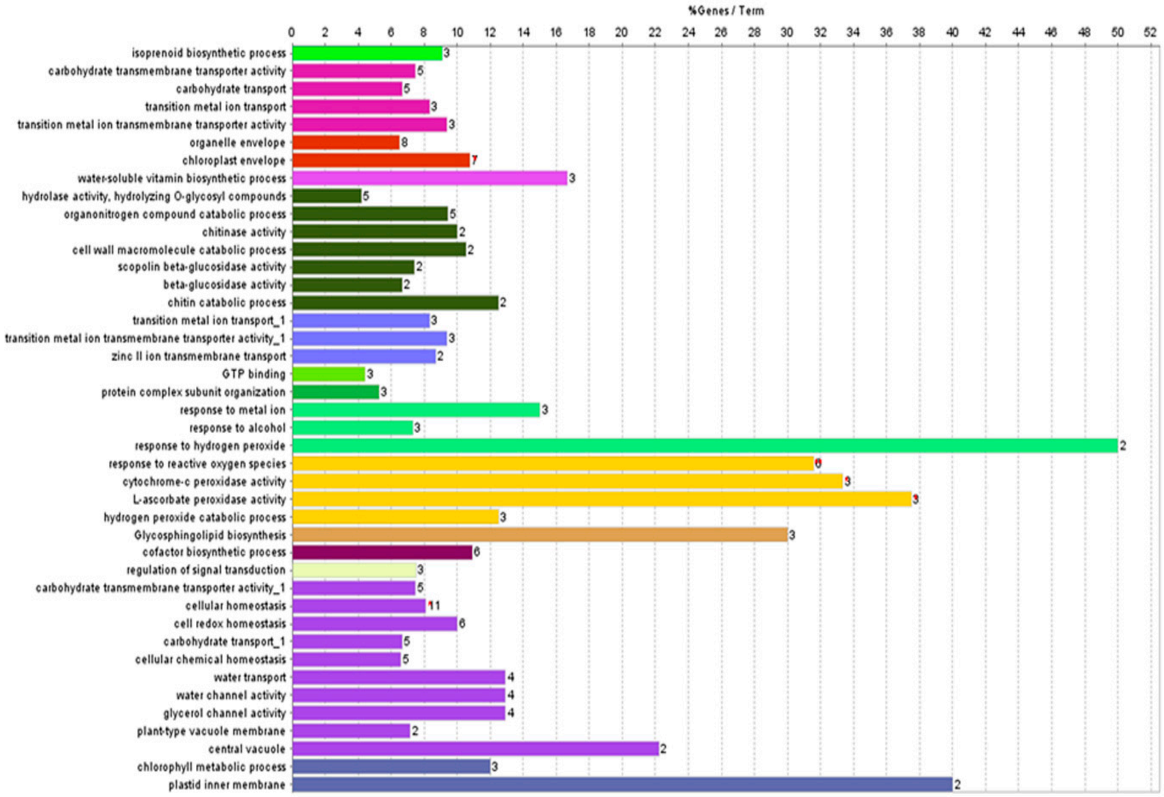


**Figure S5.** Gene ontology of the genes upregulated in transgenic rice (TR1) plants.

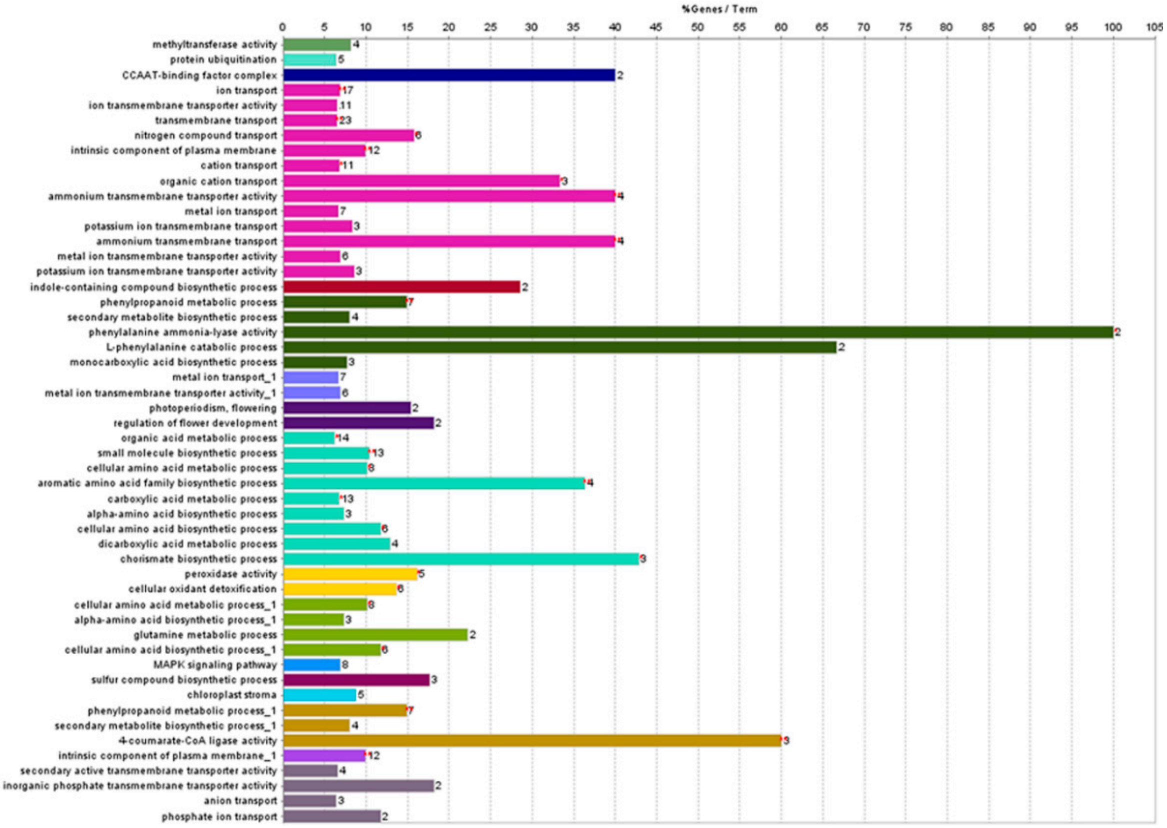


**Figure S6.** Gene ontology of the genes downregulated in transgenic rice (TR1) plants.

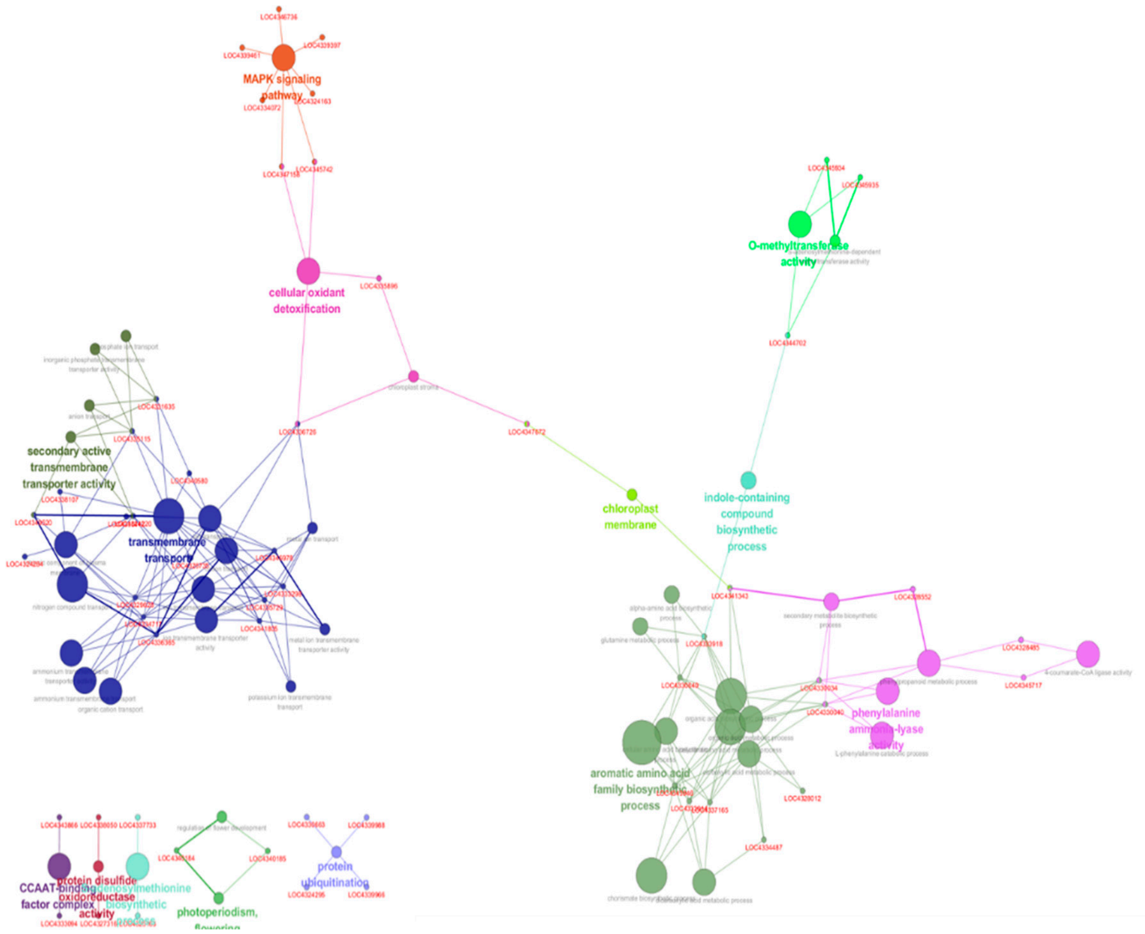


**Figure S7.** Genetic network of the genes downregulated in transgenic rice (TR1) plants.

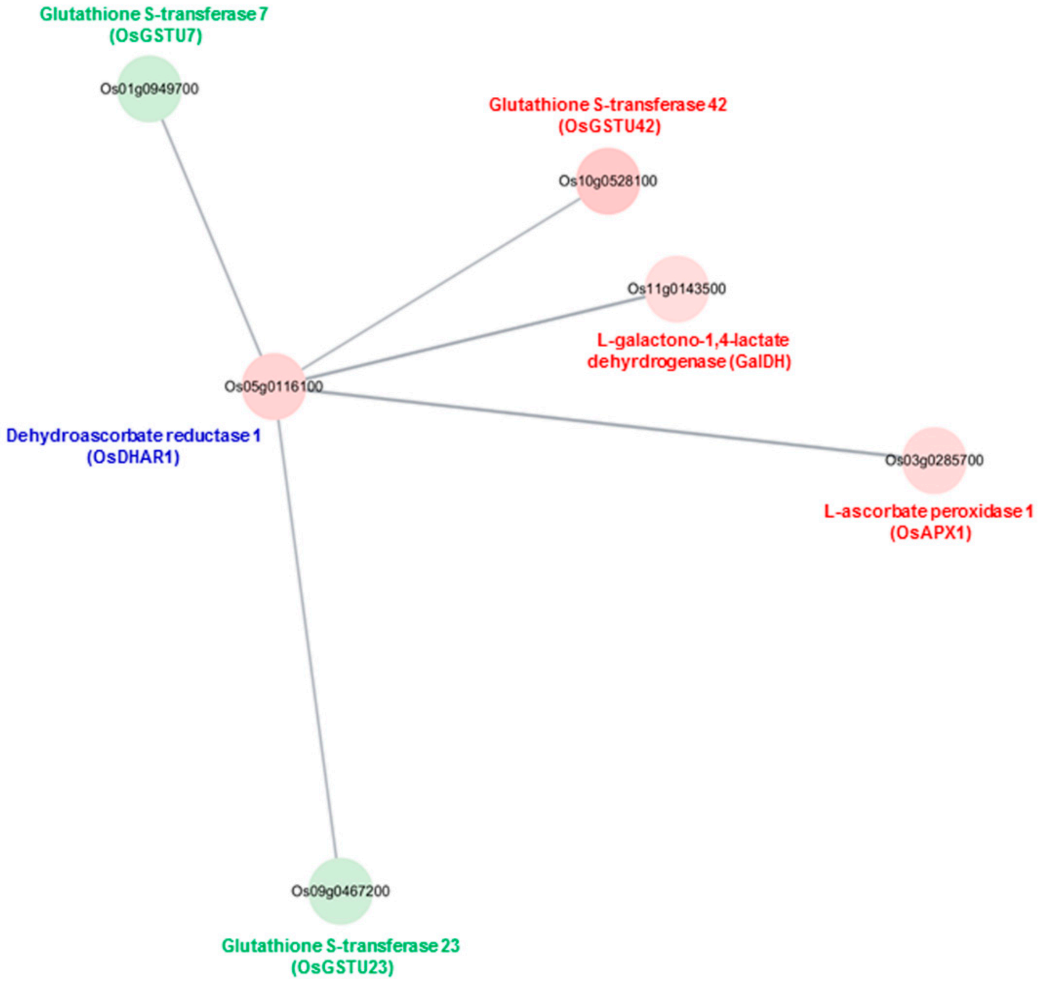


**Figure S8.** Genetic interaction map of *OsDHAR1* and other genes in transgenic rice (TR1) plants. The up- and downregulated genes are marked in red and green, respectively.

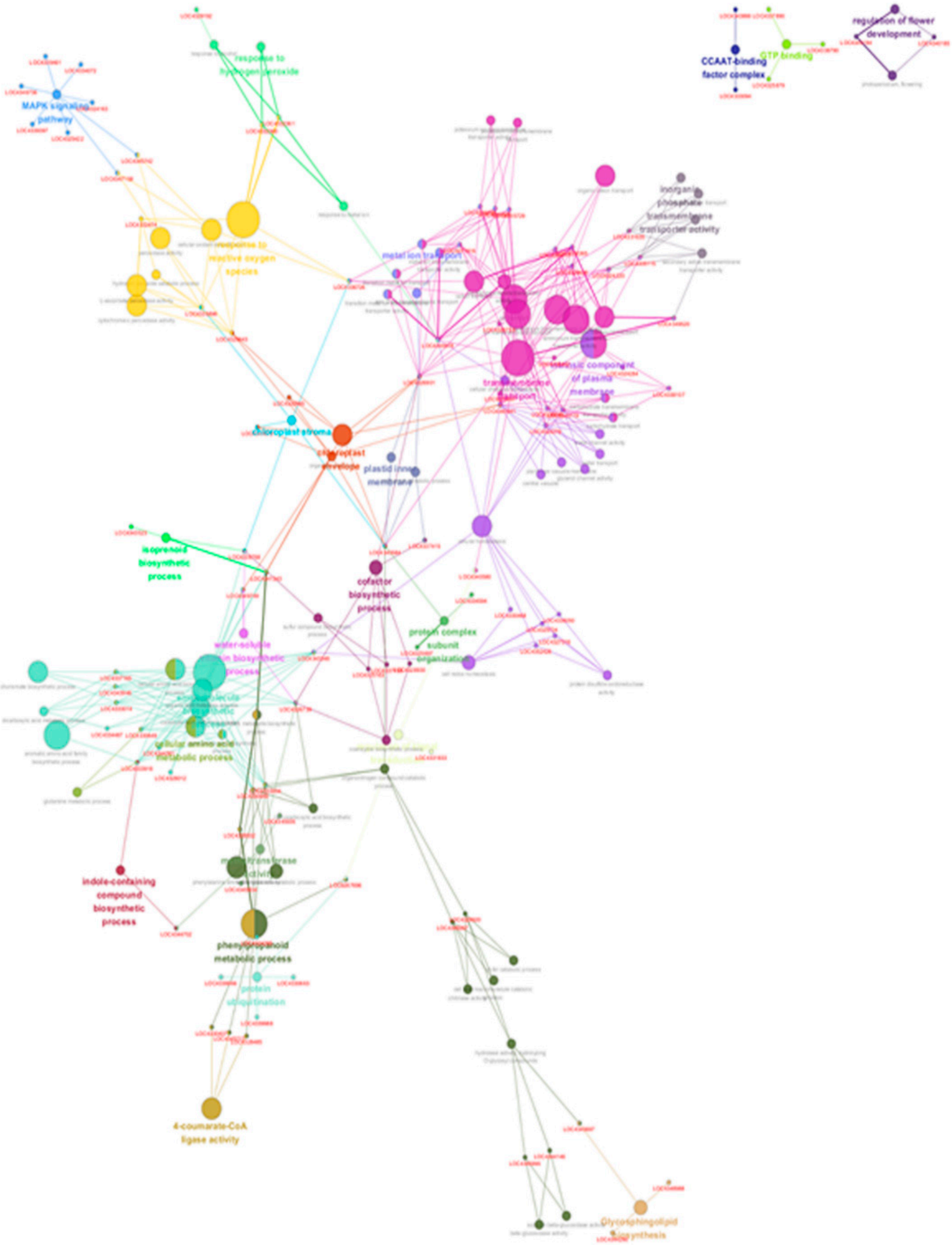


**Figure S9.** Genetic network of up- and downregulated genes in transgenic rice (TR1) plants.

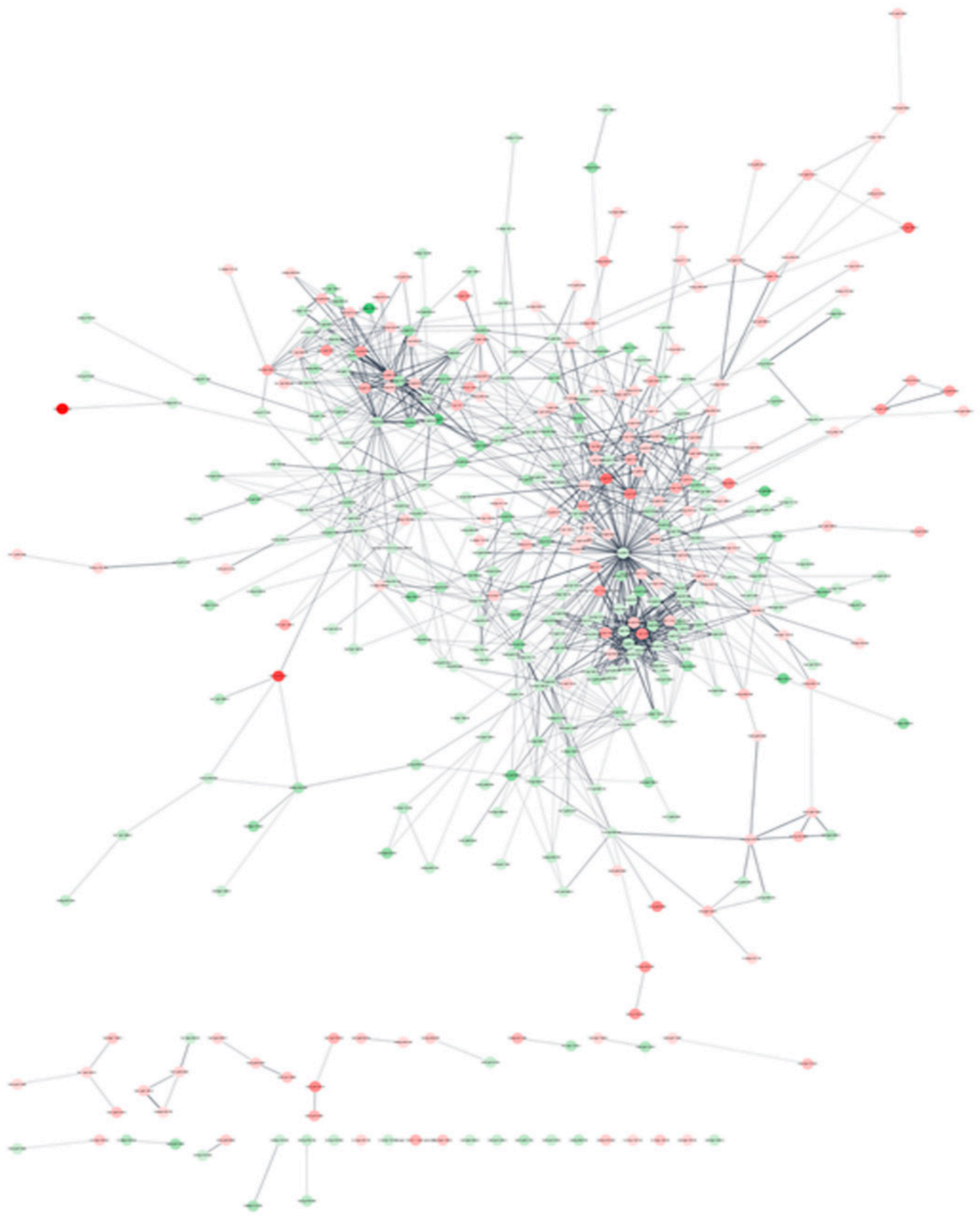

**Figure S10.** Gene to gene network of differentially expressed genes.

**Table S1.** Oligonucleotides used in the present study.

| Oligo name | Oligonucleotide sequences (5´-3´) |
|---|---|
| OsDHAR-FC | TCGCCGCCATGGGCGTGGAGGTGTGCG |
| OsDHAR-RC | CCAAGCAAGGGTACCTGCAGGCTGCATCTCCATTATTC |
| SWPA2-F1 | TGCCTTCATACGCTATTTATTTGCTTGG |
| OsDHAR-R1 | CCTTGCTCTTCAAGAACGTTGTGAAGC |
| OsDHAR-F2 | ATGGGCGTGGAGGTGTGCGTCAAGG |
| OsDHAR-R2 | CCTTGCTCTTCAAGAACGTTGTGAAGC |
| LP | AAGCGCAATTTTACAGCTGAG |
| RP | CCGTTAATAAATGGACCCTGC |
| RB | TTTCGCGATCCAGACTGAATG |
| GUS | ATGTGGAGTGAAGAGTATCAGTGTGC |
| ADA1 | GCGTAATACGACTCACTATAGCAATTAACC |
| ADA2 | GACTCACTATAGCAATTAAC |
| LB1 | GTGTGAGTAGTTCCCAGATAAGGGAATTAGG |
| LB2 | CGCTCATGTGTTGAGCATA |
| RB1 | CCCGCACCGATCGCCCTTCCCA |
| RB2 | GCAGCTTGAGCTTGGATCAGA |
| GalDH-F | TACAGGGAAGGGTTCGACTTCAGC |
| GalDH-R | AGCACATAAGTGTAGATGCTCAATG |
| Tub-F | TACCGTGCCCTTACTGTTCC |
| Tub-R | CGGTGGAATGTCACAGACAC |

Underlined nucleotides indicate the sites recognized by the restriction enzymes. Primer sequences were designed based on the information hosted on RAP-DB (http://rapdb.dna.affrc.go.jp/).

**Table S2.** Copy number and location of the *OsDHAR1* transgene.

| TR No. | Chromosome | Matching (%) | Chr_start | Chr_end | Type | Gene ID | Description |
|---|---|---|---|---|---|---|---|
| TR1 | Chr03 | 100 | 14536694 | 14536533 | Intergenic | Os03g0360000 upstream 3.008 kb | Similar to arginine decarboxylase |
| TR2 | Chr08 | 100 | 27527684 | 27527785 | Intron | Os08g0547500 | Similar to kinesin-like protein NACK1 |

| | | | | | | | |
|---|---|---|---|---|---|---|---|
| TR3 | Chr04 | 100 | 27722481 | 27722149 | Exon | Os04g0543700 | Similar to serine protease (fragment) |

**Table S3.** Genes upregulated in paddy field–grown transgenic rice (TR1) plants.

| Gene symbol | Intensity | Gene information |
|---|---|---|
| Os03g0115800 | 223.33 | Conserved hypothetical protein |
| Os11g0206000 | 210.14 | Conserved hypothetical protein |
| Os03g0115800 | 149.52 | Conserved hypothetical protein |
| Os07g0153100 | 114.97 | Hypothetical protein |
| Os01g0382000 | 38.57 | Similar to pathogenesis-related protein PRB1-2 precursor |
| Os06g0229000 | 13.64 | Similar to FtsH protease (VAR2) (Zinc dependent protease) |
| Os12g0628600 | 9.19 | Similar to thaumatin-like pathogenesis-related protein 3 precursor |
| Os11g0113700 | 7.41 | Serine/threonine protein kinase, abiotic stresses |
| Os06g0685300 | 6.84 | Similar to isoflavone reductase homolog IRL |
| Os11g0530600 | 6.46 | Similar to chalcone synthase C2 (EC 2.3.1.74) |
| Os12g0569500 | 5.98 | Thauatin, pathogenesis-related family protein |
| Os04g0249600 | 5.85 | Similar to senescence-associated protein DIN1 |
| Os01g0314800 | 5.02 | Late embryogenesis abundant protein 3 family protein |
| Os05g0160300 | 4.95 | Bifunctional inhibitor/plant lipid transfer protein/seed storage domain containing protein |
| Os01g0136100 | 4.48 | 16.9 kDa class I heat shock protein 1 |
| Os04g0612400 | 4.33 | Similar to root-specific protein; RCc2 protein |
| Os04g0612500 | 4.32 | Similar to 36.4 kDa proline-rich protein |
| Os04g0612400 | 4.20 | Protease inhibitor/seed storage/LTP family protein precursor |
| Os01g0184100 | 4.07 | Similar to 17.5 kDa class II heat shock protein |
| Os01g0368900 | 4.04 | Similar to glutaredoxin-C1; glutaredoxin 4 |
| Os01g0124000 | 3.92 | Similar to bowman birk trypsin inhibitor |
| Os03g0431200 | 3.86 | Similar to N-acetyl-glutamate synthase |
| Os10g0491000 | 3.83 | Plant basic secretory protein family protein |
| Os10g0490800 | 3.67 | Similar to secretory protein |
| Os03g0663400 | 3.55 | Similar to thaumatin-like protein; OsLP |
| Os12g0541700 | 3.52 | Similar to rapid alkalinization factor 2 |
| CB647177 | 3.13 | Photosystem II 10 kDa polypeptide, chloroplast precursor |
| Os10g0528100 | 3.06 | Similar to glutathione S-transferase GST 42; OsGSTU24 |
| Os05g0413200 | 2.89 | Beta-tubulin; OsTUB6 |
| Os03g0820500 | 2.84 | Actin depolymerizing factor 3; OsADF3 |
| Os02g0758000 | 2.77 | Similar to low molecular weight heat shock protein precursor (mitochondrial small heat shock protein 22); OsHsp24.1 |
| Os01g0279100 | 2.71 | Similar to basic leucine zipper transcription factor CAT103 |
| Os06g0625400 | 2.68 | Similar to metalloendopeptidase |

| | | |
|---|---|---|
| Os01g0186000 | 2.67 | Similar to WRKY transcription factor 10; OsWRKY10 |
| Os02g0258900 | 2.65 | Similar to molybdopterin biosynthesis CNX2 protein |
| Os04g0688100 | 2.65 | Peroxidase; Prx59 |
| Os06g0650900 | 2.62 | Heat shock protein DnaJ family protein; OsDjC56 |
| Os05g0582000 | 2.58 | Plant basic secretory protein domain containing protein |
| Os10g0548600 | 2.56 | Beta-expansin precursor; OsEXPB9 |
| Os03g0218500 | 2.52 | Similar to 70kD heat shock protein |
| BQ907184 | 2.49 | Photosystem II 10 kDa polypeptide, chloroplast precursor |
| Os03g0267000 | 2.45 | Low molecular mass heat shock protein Oshsp18.0; OsHSP18.0-CI |
| Os01g0232000 | 2.44 | Major intrinsic protein family protein; probable aquaporin TIP4.3 (OsTIP4;3) |
| Os05g0116100 | 2.44 | Dehydroascorbate reductase 1; OsDHAR1 |
| Os03g0843800 | 2.43 | Methyl chloride transferase; harmless to ozone layer 1 (OsHOL1) |
| Os04g0691900 | 2.42 | Chaperonin Cpn60/TCP-1 family protein |
| Os03g0180900 | 2.42 | Jasmonate ZIM-domain protein 2; OsJAZ2 |
| Os03g0848700 | 2.38 | Brown planthopper resistance-14; Bph14 |
| Os01g0111100 | 2.38 | Cyclophilin-like domain containing protein; OsCYP26-2 |
| Os01g0838350 | 2.37 | Phosphate-limitation inducible gene 1; OsPI1 |
| Os02g0629200 | 2.36 | Probable aquaporin PIP2-2; OsPIP2;2 |
| Os01g0583300 | 2.31 | Zinc finger, C2H2, LYAR-type domain containing protein; Os_F0727 |
| Os03g0161900 | 2.31 | Heat stress transcription factor A2d; HSfA2d |
| Os04g0345400 | 2.30 | Terpene synthase 24; OsTPS24 |
| Os03g0782500 | 2.30 | Phytochrome-interacting factor-like protein 1; OsPIL1 |
| Os10g0506100 | 2.25 | Heavy metal transport/detoxification protein domain containing protein |
| AK100788.1 | 2.24 | ABA/WDS induced protein |
| Os01g0939500 | 2.23 | Similar to eukaryotic peptide chain release factor subunit 1-2 (eRF1-2) |
| Os02g0553200 | 2.23 | Ascorbate peroxidase 8; OsAPx8 |
| Os04g0423400 | 2.20 | Abiotic stress responsive 2 (OsASR2); ABA-, stress and ripening-induced protein 4 (OsASR4) |
| Os01g0117900 | 2.20 | Similar to nodulin-like protein |
| Os11g0222225 | 2.18 | Serpin domain containing protein |
| Os02g0707000 | 2.17 | Monodehydroascorbate reductase 1; OsMDHAR1 |
| Os01g0284700 | 2.17 | Similar to peptidyl-prolyl cis-trans isomerase; OsCYP20-3 |
| Os11g0592000 | 2.15 | Pathogenesis-related protein 4c; OsPR4c |
| Os08g0479300 | 2.13 | Cyclin, A/B/D/E domain containing protein |
| Os03g0285700 | 2.11 | L-ascorbate peroxidase 1; OsAPX1 |
| Os03g0745000 | 2.10 | Heat stress transcription factor A2a; HSfA2 |
| Os01g0232000 | 2.09 | Probable aquaporin TIP4.3 |
| Os05g0515900 | 2.08 | Similar to heat- and acid-stable phosphoprotein |
| Os05g0247100 | 2.08 | Drought-induced protein 3; DIP3 |
| Os02g0753800 | 2.07 | Similar to annexin p35; OsANN2 |
| Os12g0538700 | 2.07 | Glutaredoxin 29; OsGRX29 |
| Os06g0158900 | 2.04 | Similar to multidrug-resistance associated protein 3 |

| | | |
|---|---|---|
| Os03g0163300 | 2.03 | Glutathione reductase 1 |
| Os01g0175600 | 2.02 | Boron (B) transporter; OsBOR2 |
| Os08g0112700 | 2.02 | Similar to TAGL12 transcription factor |
| Os11g0143500 | 2.00 | Similar to L-galactono-1,4-lactone dehydrogenase; GalDH |

**Table S4.** ClueGO classification of the genes upregulated in transgenic (TR1) plants.

| GO ID | GO Term | Ontology source | Associated genes |
|---|---|---|---|
| GO:0000302 | Response to reactive oxygen species | BP | LOC4329643, LOC4332360, LOC4332361, LOC4332474 |
| GO:0004130 | Cytochrome-c peroxidase activity | MF | LOC4329643, LOC4332474 |
| GO:0016688 | L-ascorbate peroxidase activity | MF | LOC4329643, LOC4332474 |
| GO:0042542 | Response to hydrogen peroxide | BP | LOC4332360, LOC4332361 |
| GO:0042744 | Hydrogen peroxide catabolic process | BP | LOC4329643, LOC4332474 |
| GO:0098869 | Cellular oxidant detoxification | BP | LOC4329643, LOC4332474 |
| GO:0045471 | Response to ethanol | BP | LOC4332360, LOC4332361 |
| GO:0046686 | Response to cadmium ion | BP | LOC4332360, LOC4332361 |
| GO:0046688 | Response to copper ion | BP | LOC4332360, LOC4332361 |
| GO:0051188 | Cofactor biosynthetic process | BP | LOC4326739, LOC4326901, LOC4328930, LOC4337415 |
| GO:0009108 | Coenzyme biosynthetic process | BP | LOC4326739, LOC4328930 |
| GO:0031967 | Organelle envelope | CC | LOC4326901, LOC4329643, LOC4333985, LOC4340941, LOC4343584, LOC4349749 |
| GO:0015994 | Chlorophyll metabolic process | BP | LOC4326901, LOC4337415, LOC4343584 |
| GO:0009528 | Plastid inner membrane | CC | LOC4326901, LOC4343584 |
| GO:0009941 | Chloroplast envelope | CC | LOC4326901, LOC4329643, LOC4333985, LOC4340941, LOC4343584 |
| GO:0005525 | GTP binding | MF | LOC4325879, LOC4337890, LOC4338790 |
| GO:0008299 | Isoprenoid biosynthetic process | BP | LOC4338768, LOC4343523 |
| GO:0009966 | Regulation of signal transduction | BP | LOC4331833, LOC9267696 |

| | | | |
|---|---|---|---|
| GO:0004553 | Hydrolase activity, hydrolyzing O-glycosyl compounds | MF | LOC4329920, LOC4343697, LOC4344146, LOC4345995, LOC4349267 |
| GO:1901565 | Organonitrogen compound catabolic process | BP | LOC4329920, LOC4343584, LOC4349267 |
| GO:0004568 | Chitinase activity | MF | LOC4329920, LOC4349267 |
| GO:0016998 | Cell wall macromolecule catabolic process | BP | LOC4329920, LOC4349267 |
| GO:0102483 | Scopolin beta-glucosidase activity | MF | LOC4344146, LOC4345995 |
| GO:0008422 | Beta-glucosidase activity | MF | LOC4344146, LOC4345995 |
| GO:0006032 | Chitin catabolic process | BP | LOC4329920, LOC4349267 |
| GO:0019725 | Cellular homeostasis | BP | LOC4324276, LOC4325734, LOC4330049, LOC4330468, LOC4340941, LOC4343119, LOC4343348, LOC4352426 |
| GO:0045454 | Cell redox homeostasis | BP | LOC4325734, LOC4330468, LOC4343348, LOC4352426 |
| GO:0006833 | Water transport | BP | LOC4324276, LOC4330049, LOC4340941, LOC4343119 |
| GO:0015250 | Water channel activity | MF | LOC4324276, LOC4330049, LOC4340941, LOC4343119 |
| GO:0009992 | Cellular water homeostasis | BP | LOC4324276, LOC4330049, LOC4340941, LOC4343119 |
| GO:0015254 | Glycerol channel activity | MF | LOC4324276, LOC4330049, LOC4340941, LOC4343119 |
| GO:0015793 | Glycerol transport | BP | LOC4324276, LOC4330049, LOC4340941, LOC4343119 |
| GO:0009705 | Plant-type vacuole membrane | CC | LOC4324276, LOC4340941 |
| GO:0042807 | Central vacuole | CC | LOC4324276, LOC4340941 |
| GO:0044283 | Small molecule biosynthetic process | BP | LOC4326739, LOC4338768, LOC4343348, LOC4344361, LOC4349749 |
| GO:0042364 | Water-soluble vitamin biosynthetic process | BP | LOC4326739, LOC4338768, LOC4349749 |
| GO:0019725 | Cellular homeostasis | BP | LOC4324276, LOC4325734, LOC4330049, LOC4330468, LOC4340941, LOC4343119, LOC4343348, LOC4352426 |
| GO:0045454 | Cell redox homeostasis | BP | LOC4325734, LOC4330468, LOC4343348, LOC4352426 |

| | | | | | |
|---|---|---|---|---|---|
| GO:0015035 | Protein disulfide oxidoreductase activity | MF | LOC4325734, | LOC4352426 | |
| GO:0005385 | Zinc ion transmembrane transporter activity | MF | LOC4326901, | LOC9271816 | |
| GO:0071577 | Zinc II ion transmembrane transport | BP | LOC4326901, | LOC9271816 | |
| GO:0071822 | Protein complex subunit organization | BP | LOC4325697, | LOC4334594, | LOC4343584 |
| GO:0000603 | Glycosphingolipid biosynthesis | KEGG | LOC4343697, | LOC4344290, | LOC4348988 |

BP, biological process; MF, molecular function; CC, cellular component.
LOC number was identified by Quick GO (https://www.ebi.ac.uk/QuickGO/) which is a fast browser for Gene Ontology terms and annotations.

**Table S5.** Genes downregulated in paddy field–grown transgenic rice (TR1) plants.

| Gene symbol | Intensity | Gene information |
|---|---|---|
| Os11g0188850 | 0.01 | RNA-directed DNA polymerase |
| AK062276.1 | 0.01 | F-box domain containing protein |
| Os11g0182500 | 0.01 | Ankyrin repeat domain containing protein; OsPIANK1 |
| Os06g0185400 | 0.05 | Conserved hypothetical protein |
| CB656443 | 0.06 | Cysteine protease 1 precursor |
| Os02g0582600 | 0.10 | Heavy metal transport/detoxification protein domain containing protein |
| Os02g0187800 | 0.17 | Cinnamyl alcohol dehydrogenase |
| Os06g0579200 | 0.19 | Low-affinity cation transporter |
| Os09g0417600 | 0.23 | WRKY transcription factor 76; OsWRKY76 |
| Os01g0816700 | 0.24 | Similar to L-ascorbate oxidase homolog precursor |
| Os08g0157500 | 0.25 | Quercetin 3-O-methyltransferase 1; COMT |
| Os09g0532900 | 0.27 | Transcription factor MYB42/85; OsMYB42/85 |
| Os04g0675400 | 0.27 | DnaJ domain protein C45; OsDjC45 |
| Os05g0322900 | 0.28 | Similar to WRKY transcription factor 45; OsWRKY45 |
| Os09g0456800 | 0.28 | Similar to heat stress transcription factor Spl7 (Heat shock transcription factor) (Heat shock factor RHSF10) |
| Os03g0179400 | 0.29 | Drought-inducible receptor-like cytoplasmic kinase, Drought tolerance, Regulator of grain yield |
| Os04g0434800 | 0.29 | Similar to thylakoid-bound ascorbate peroxidase; OsAPx7 |
| Os11g0199700 | 0.30 | Similar to VHS and GAT domain protein; similar to seed protein B32E |
| Os06g0157700 | 0.30 | Florigen, heading date; flowering locus T |
| Os06g0111600 | 0.30 | Serine/threonine protein kinase-related domain containing protein; OsWAK11 |
| Os10g0552400 | 0.30 | Zinc finger, RING/FYVE/PHD-type domain containing protein; OsPUB67 |

| | | |
|---|---|---|
| Os11g0490900 | 0.32 | Similar to WRKY transcription factor 72; OsWRKY72 |
| Os04g0675400 | 0.32 | Similar to chaperone protein dnaJ; OsDjC45 |
| Os01g0134700 | 0.32 | Calmodulin binding protein-like family protein |
| LOC_Os01g06900 | 0.32 | Verticillium wilt disease resistance protein Ve2 |
| Os03g0187400 | 0.33 | Zinc finger, FYVE/PHD-type domain containing protein |
| Os06g0232300 | 0.35 | Similar to auxin transport protein REH1 |
| Os01g0289666 | 0.35 | WRKY9 |
| Os09g0532000 | 0.36 | TonB box, conserved site domain containing protein; stay green leaf (t), stay green, staying green during senescence |
| Os11g0597700 | 0.36 | Similar to resistant protein |
| Os07g0683200 | 0.37 | Similar to NAC domain transcription factor; ONAC103 |
| Os01g0141000 | 0.37 | APETALA2/ethylene-responsive element binding protein 129; AP2/EREBP#129 |
| Os06g0671600 | 0.37 | Small auxin-up RNA 26; OsSAUR26 |
| Os01g0194600 | 0.37 | Glutaredoxin 2; OsGRX2 |
| Os01g0971800 | 0.37 | Similar to two-component response regulator ARR11 |
| Os01g0178900 | 0.37 | Similar to symbiosis-related disease resistance protein |
| Os03g0626700 | 0.38 | Multi antimicrobial extrusion protein MatE family protein |
| Os07g0680400 | 0.39 | DNA-binding WRKY domain containing protein; OsWRKY47 |
| Os06g0143100 | 0.39 | Similar to cadmium tolerant 1 |
| Os09g0313600 | 0.40 | Disease resistance protein domain containing protein |
| Os02g0303500 | 0.40 | Eggshell protein family protein |
| Os11g0184900 | 0.41 | Similar to OsNAC5 protein |
| Os11g0126900 | 0.41 | NAC-domain protein, drought tolerance |
| Os06g0157500 | 0.41 | Florigen, flowering time, long-day promotion |
| Os05g0120700 | 0.42 | ATPase, P-type, K/Mg/Cd/Cu/Zn/Na/Ca/Na/H-transporter family protein |
| Os07g0665200 | 0.43 | Cu/Zn-superoxide dismutase; Cu/Zn-SOD |
| Os01g0665500 | 0.43 | Similar to WRKY transcription factor 16; OsWRKY16 |
| Os05g0439000 | 0.43 | Similar to RING-H2 finger protein ATL1O |
| Os05g0198200 | 0.43 | Glutaredoxin 16; OsGRX16 |
| Os02g0738200 | 0.43 | Zinc finger, RING/FYVE/PHD-type domain containing protein |
| LOC_Os03g25150 | 0.43 | Flavonoid 3,5-hydroxylase 2 |
| Os03g0341600 | 0.44 | Pollen Ole e 1 allergen/extensin domain containing protein |
| Os06g0125800 | 0.44 | Zinc finger, RING/FYVE/PHD-type domain containing protein |
| Os04g0418500 | 0.44 | Similar to photoperiod responsive protein |
| Os03g0182800 | 0.44 | Similar to ethylene responsive element binding factor3 (OsERF3); EREBP transcription factor |
| Os01g0135900 | 0.44 | Similar to cytosolic class I small heat shock protein 3C; OsHsp17.9B |
| Os03g0787000 | 0.44 | Similar to syntaxin |
| Os06g0218300 | 0.44 | Zinc finger, RING-type domain containing protein |
| Os05g0549800 | 0.44 | Similar to DNA-binding protein RAV1; APETALA2/ethylene-responsive element binding protein 96 (AP2/EREBP#096) |

| | | |
|---|---|---|
| Os02g0571100 | 0.44 | Terpenoid synthase domain containing protein |
| Os05g0530400 | 0.45 | Heat stress transcription factor Spl7 (Heat shock transcription factor) (Heat shock factor RHSF10); HSF10 |
| Os07g0107300 | 0.45 | Plant disease resistance response protein family protein |
| Os06g0614100 | 0.45 | bZIP transcription factor, bZIP-1 domain containing protein; OsbZIP49 |
| Os01g0974400 | 0.45 | Zinc finger, RING/FYVE/PHD-type domain containing protein; OsRFPHC-7 |
| LOC_Os09g04430 | 0.45 | Lactoylglutathione lyase |
| Os01g0563000 | 0.45 | Peptidyl-prolyl cis-trans isomerase, FKBP-type domain containing protein; OsFKBP73 |
| Os01g0949700 | 0.45 | Glutathione S-transferase, C-terminal domain containing protein; OsGSTU7 |
| Os01g0723100 | 0.45 | Senescence-associated family protein |
| Os01g0933900 | 0.45 | Similar to glutathione transferase III(B); OsGSTF4 |
| Os01g0542700 | 0.45 | bZIP transcription factor, bZIP-1 domain containing protein; OsbZIP04 |
| Os12g0105600 | 0.45 | Similar to phosphate starvation regulator protein (regulatory protein of P- starvation acclimation response Psr1). |
| Os03g0411100 | 0.45 | Similar to Nuclear Y/CCAAT-box binding factor A subunit NF-YA; OsHAP2E, NF-YA, CBF-B |
| Os05g0553400 | 0.45 | Similar to Myb-related transcription factor-like protein (MYB transcription factor); OsMYB55, OsMYB-2 |
| Os08g0386200 | 0.46 | WRKY transcription factor 69; OsWRKY69 |
| Os01g0949700 | 0.46 | Glutathione S-transferase, C-terminal domain containing protein; OsGSTU7 |
| Os02g0578400 | 0.46 | Photosystem II oxygen evolving complex protein PsbQ family protein |
| Os03g0718800 | 0.46 | Similar to physical impedance induced protein |
| Os06g0701600 | 0.46 | Monovalent cation transporter, Na+ and K+ transport; OsHKT9, OsHKT2;4 |
| Os09g0524800 | 0.46 | Apoptosis regulator Bcl-2 protein, BAG domain containing protein; OsBAG1 |
| Os03g0835900 | 0.46 | Similar to ferredoxin III, chloroplast precursor (Fd III) |
| Os03g0259100 | 0.46 | Similar to basic blue protein (cusacyanin) (plantacyanin) (CBP) |
| Os09g0467200 | 0.47 | Similar to glutathione S-transferase GST 23 |
| Os05g0557700 | 0.47 | Conserved hypothetical protein; leaf tip necrosis1, phosphate overaccumulator 2 |
| Os10g0159800 | 0.48 | Alcohol dehydrogenase superfamily, zinc-containing protein |
| Os02g0105400 | 0.48 | Similar to L-lactate dehydrogenase A |
| Os07g0608200 | 0.49 | Similar to CCAAT-binding transcription factor subunit B; CBF-B |
| Os05g0537100 | 0.49 | WRKY transcription factor 10 |
| Os01g0246700 | 0.49 | Similar to WRKY transcription factor 1; OsWRKY1 |
| Os06g0292400 | 0.49 | Embryogenesis transmembrane protein |
| Os01g0225000 | 0.49 | Mitochondrial carrier protein domain containing protein |

Intensity is represented as a $Log_2$ ratio.

**Table S6.** ClueGO classification of genes downregulated in transgenic (TR1) plants.

| GO ID | GO Term | Ontology source | Associated genes |
|---|---|---|---|
| GO:0006082 | Organic acid metabolic process | BP | LOC4328012, LOC4330034, LOC4330040, LOC4330649, LOC4333014, LOC4333918, LOC4334487, LOC4337165, LOC4341343, LOC4343946 |
| GO:0016053 | Organic acid biosynthetic process | BP | LOC4330034, LOC4330040, LOC4330649, LOC4333014, LOC4333918, LOC4337165, LOC4341343, LOC4343946 |
| GO:0006520 | Cellular amino acid metabolic process | BP | LOC4330034, LOC4330040, LOC4330649, LOC4333014, LOC4333918, LOC4337165, LOC4343946 |
| GO:0009073 | Aromatic amino acid family biosynthetic process | BP | LOC4333014, LOC4333918, LOC4337165, LOC4343946 |
| GO:0019752 | Carboxylic acid metabolic process | BP | LOC4328012, LOC4330034, LOC4330040, LOC4330649, LOC4333014, LOC4333918, LOC4334487, LOC4337165, LOC4343946 |
| GO:1901607 | Alpha-amino acid biosynthetic process | BP | LOC4330649, LOC4333918 |
| GO:0006541 | Glutamine metabolic process | BP | LOC4330649, LOC4333918 |
| GO:0008652 | Cellular amino acid biosynthetic process | BP | LOC4330649, LOC4333014, LOC4333918, LOC4337165, LOC4343946 |
| GO:0043648 | Dicarboxylic acid metabolic process | BP | LOC4333014, LOC4334487, LOC4337165, LOC4343946 |
| GO:0009423 | Chorismate biosynthetic process | BP | LOC4333014, LOC4337165, LOC4343946 |
| GO:0008171 | O-methyltransferase activity | MF | LOC4344702, LOC4345934, LOC4345935 |
| GO:0008757 | S-adenosylmethionine-dependent methyltransferase activity | MF | LOC4344702, LOC4345934, LOC4345935 |
| GO:0006556 | S-adenosylmethionine biosynthetic process | BP | LOC4325163, LOC4337733 |
| GO:0006811 | Ion transport | BP | LOC4324220, LOC4329628, LOC4331635, LOC4333298, LOC4334717, LOC4335115, LOC4335729, LOC4336365, LOC4336726, LOC4341805, LOC4346978 |

| | | | |
|---|---|---|---|
| GO:0015075 | Ion transmembrane transporter activity | MF | LOC4324220, LOC4329628, LOC4333298, LOC4334717, LOC4335729, LOC4336365, LOC4341805, LOC4346978 |
| GO:0055085 | Transmembrane transport | BP | LOC4324220, LOC4326735, LOC4329628, LOC4331635, LOC4333298, LOC4334717, LOC4335115, LOC4335729, LOC4336365, LOC4336442, LOC4338107, LOC4340580, LOC4341805, LOC4346978, LOC4349520 |
| GO:0071705 | Nitrogen compound transport | BP | LOC4324220, LOC4329628, LOC4334717, LOC4336365, LOC4349520 |
| GO:0031226 | Intrinsic component of plasma membrane | CC | LOC4324220, LOC4324284, LOC4329628, LOC4334717, LOC4335115, LOC4336365, LOC4338107, LOC4349520 |
| GO:0006812 | Cation transport | BP | LOC4329628, LOC4333298, LOC4334717, LOC4335729, LOC4336365, LOC4336726, LOC4341805, LOC4346978 |
| GO:0034220 | Ion transmembrane transport | BP | LOC4324220, LOC4329628, LOC4333298, LOC4334717, LOC4335729, LOC4336365, LOC4341805, LOC4346978 |
| GO:0015695 | Organic cation transport | BP | LOC4329628, LOC4334717, LOC4336365 |
| GO:0008519 | Ammonium transmembrane transporter activity | MF | LOC4329628, LOC4334717, LOC4336365 |
| GO:0030001 | Metal ion transport | BP | LOC4333298, LOC4335729, LOC4336726, LOC4341805, LOC4346978 |
| GO:0071805 | Potassium ion transmembrane transport | BP | LOC4333298, LOC4335729, LOC4341805 |
| GO:0072488 | Ammonium transmembrane transport | BP | LOC4329628, LOC4334717, LOC4336365 |
| GO:0046873 | Metal ion transmembrane transporter activity | MF | LOC4333298, LOC4335729, LOC4341805, LOC4346978 |
| GO:0098869 | Cellular oxidant detoxification | BP | LOC4335896, LOC4336726, LOC4345742, LOC4347158 |
| GO:0009570 | Chloroplast stroma | CC | LOC4335896, LOC4336726, LOC4347672 |
| GO:0004016 | MAPK signaling pathway | KEGG | LOC4324163, LOC4334072, LOC4339397, LOC4339461, LOC4345742, LOC4346736, LOC4347158 |
| GO:0009698 | Phenylpropanoid metabolic process | BP | LOC4328485, LOC4328552, LOC4330034, LOC4330040, LOC4345717 |

| | | | |
|---|---|---|---|
| GO:0044550 | Secondary metabolite biosynthetic process | BP | LOC4328552, LOC4330034, LOC4330040, LOC4341343 |
| GO:0016207 | 4-coumarate-CoA ligase activity | MF | LOC4328485, LOC4345717 |
| GO:0045548 | Phenylalanine ammonia-lyase activity | MF | LOC4330034, LOC4330040 |
| GO:0006559 | L-phenylalanine catabolic process | BP | LOC4330034, LOC4330040 |
| GO:0015035 | Protein disulfide oxidoreductase activity | MF | LOC4327316, LOC4338050 |
| GO:0015291 | Secondary active transmembrane transporter activity | MF | LOC4324220, LOC4331635, LOC4335115, LOC4349520 |
| GO:0005315 | Inorganic phosphate transmembrane transporter activity | MF | LOC4331635, LOC4335115 |
| GO:0006820 | Anion transport | BP | LOC4324220, LOC4331635, LOC4335115 |
| GO:0006817 | Phosphate ion transport | BP | LOC4331635, LOC4335115 |
| GO:0016567 | Protein ubiquitination | BP | LOC4324295, LOC4330663, LOC4339966, LOC4339988 |
| GO:0016602 | CCAAT-binding factor complex | CC | LOC4333094, LOC4343866 |
| GO:0031969 | Chloroplast membrane | CC | LOC4341343, LOC4347672 |
| GO:0042435 | Indole-containing compound biosynthetic process | BP | LOC4333918, LOC4344702 |
| GO:0048573 | Photoperiodism, flowering | BP | LOC4340184, LOC4340185 |
| GO:0009909 | Regulation of flower development | BP | LOC4340184, LOC4340185 |

BP, biological process; MF, molecular function; CC, cellular component

LOC number was identified using Quick GO (https://www.ebi.ac.uk/QuickGO/), which is a fast browser for Gene Ontology terms and annotations.

**Table S7.** Functional classification of genes differentially expressed in transgenic rice (TR1) plants.

| Functional category | Gene count | Related gene |
|---|---|---|
| Apoptotic process | 13 | Os01g0229400, Os11g0598500, Os03g0254000, Os03g0848700, Os03g0848700, Os10g0124300, Os12g0512400, Os09g0524800, Os11g0579200, Os11g0598300, Os02g0282000, Os11g0226933, Os11g0598500, Os09g0313600 |
| Cell cycle | 2 | Os12g0143800, Os08g0479300 |

| | | |
|---|---|---|
| Cell growth | 6 | Os07g0679300, Os08g0509200, Os05g0548900, Os07g0148800, Os01g0142800, Os07g0690900 |
| Biological regulation | 137 | Os01g0106400, Os07g0679300, Os07g0509800, Os01g0229400, Os11g0530600, Os03g0355900, Os01g0124000, Os11g0113700, Os07g0558500, Os03g0640800, Os02g0257300, Os05g0161500, Os11g0222225, Os05g0521300, Os07g0509800, Os04g0683700, Os05g0506000, Os03g0745000, Os03g0764600, Os09g0426800, Os12g0438600, Os05g0575000, Os06g0158900, Os01g0911300, Os05g0217800, etc. |
| Biosynthetic process | 128 | Os08g0434100, Os10g0502400, Os03g0856500, Os03g0856500, Os06g0685300, Os07g0509800, Os09g0451400, Os04g0678700, Os11g0530600, Os12g0143800, Os03g0797400, Os03g0640800, Os07g0689600, Os07g0691200, Os07g0691200, Os11g0644800, Os02g0194700, Os02g0257300, Os11g0222225, Os05g0548900, Os01g0812800, Os03g0423300, Os03g0431200, Os11g0491400, Os01g0225400, Os03g0850400, Os09g0294000, etc. |
| Cell adhesion | 8 | Os05g0453300, Os07g0148800, Os03g0764600, Os11g0667700, Os01g0911300, Os11g0199700, Os07g0511400, Os09g0364400 |
| Cell differentiation | 10 | Os07g0679300, Os07g0124100, Os06g0157700, Os06g0157500, Os08g0509200, Os05g0548900, Os07g0148800, Os09g0426800, Os01g0142800, Os02g0122600 |
| Cell development | 6 | Os07g0679300, Os08g0509200, Os05g0548900, Os07g0148800, Os01g0142800, Os02g0122600 |
| Lipid metabolic process | 39 | Os06g0685300, Os07g0679300, Os11g0530600, Os03g0355900, Os07g0558500, Os01g0717000, Os03g0700700, Os09g0513100, Os02g0194700, Os02g0257300, Os05g0548900, Os03g0423300, Os02g0589000, Os02g0596000, Os01g0757200, Os07g0546000, Os09g0426800, Os01g0369900, Os02g0571100, Os07g0616200, Os03g0261100, Os05g0363200, Os10g0140300, Os10g0533500, Os07g0598000, Os03g0356582, Os06g0694200, Os06g0569500, Os07g0190000, Os06g0600400, Os05g0408900, Os10g0493600, Os08g0441500, Os07g0412100, Os07g0162100, Os06g0685300 |
| Transport | 102 | Os08g0434100, Os06g0685300, Os07g0679300, Os01g0229400, Os05g0160300, Os11g0530600, Os07g0558500, Os07g0689600, Os02g0257300, Os03g0638200, Os03g0638200, Os06g0264500, Os02g0596000, Os01g0232000, Os04g0683700, Os06g0566300, Os12g0438600, Os01g0825800, Os04g0612400, Os04g0612400, Os04g0612500, Os04g0612500, Os09g0537700, etc. |
| Translation | 10 | Os03g0856500, Os03g0856500, Os01g0812800, Os01g0185200, Os09g0265800, Os09g0294000, Os01g0939500, Os02g0529700, Os03g0816200, Os04g0573200 |
